# Impact of probiotics-derived extracellular vesicles on livestock gut barrier function

**DOI:** 10.1186/s40104-024-01102-8

**Published:** 2024-11-07

**Authors:** Yuhan Zhang, Mengzhen Song, Jinping Fan, Xuming Guo, Shiyu Tao

**Affiliations:** College of Animal Sciences and Technology, Huazhong Agricultural University, No. 1 Shizishan Street, Hongshan District, Wuhan, Hubei Province 430070 China

**Keywords:** Extracellular vesicles, Intestinal barrier, Livestock production, Probiotics

## Abstract

Probiotic extracellular vesicles (pEVs) are biologically active nanoparticle structures that can regulate the intestinal tract through direct or indirect mechanisms. They enhance the intestinal barrier function in livestock and poultry and help alleviate intestinal diseases. The specific effects of pEVs depend on their internal functional components, including nucleic acids, proteins, lipids, and other substances. This paper presents a narrative review of the impact of pEVs on the intestinal barrier across various segments of the intestinal tract, exploring their mechanisms of action while highlighting the limitations of current research. Investigating the mechanisms through which probiotics operate via pEVs could deepen our understanding and provide a theoretical foundation for their application in livestock production.

## Introduction

The majority of gut microorganisms are bacteria [1]. Probiotics are microorganisms that provide health benefits to the host when provided in sufficient numbers [2–4]. Common intestinal probiotics include *Lactobacillus*, *Bifidobacterium*, *Streptococcus*, and certain strains of *Escherichia coli* [5–7]. Intestinal probiotics play important roles in maintaining the balance of intestinal flora, promoting nutrient absorption, enhancing immunity, and alleviating constipation and diarrhea [8, 9].

Probiotics can exert their effects through biologically active nanoparticle structures released as probiotic extracellular vesicles (pEVs), which are currently a focus of emerging research. pEVs, which are nanoparticles encapsulated by phospholipid bilayers, are derived from probiotics [10–12]. Most of these vesicles with sizes ranging from 30 to 200 nm contain molecules such as proteins, nucleic acids [13, 14], and lipids [15]. Research revealing that *Vibrio cholerae* can produce extracellular vesicles was published as early as 1967 [16]. At that time, many scholars considered these vesicles to be insignificant. However, subsequent studies revealed that pEVs play a crucial structural role in bacteria [17–19]. Their regulatory effects on the intestinal tract extend beyond their traditional roles as bacterial by-products [20]. pEVs transport internal molecules over long distances in a concentrated, protected, and targeted manner, serving various functions such as nutrient acquisition, biofilm formation, delivery of virulence factors, and immunomodulation [18, 21–35]. Imbalanced production of pEVs may lead to intestinal inflammation, infections, metabolic disorders, and cancer [36–38]. Therefore, conducting a comprehensive analysis of the impact of pEVs on the intestinal barrier is essential for understanding the mechanisms underlying gut microbial community-host interactions. This analysis could help uncover novel functions and potential applications for probiotics and facilitate the development of new strategies to prevent and treat intestinal diseases in livestock and poultry. Ultimately, these efforts aim to enhance the economic outcomes of animal husbandry practices.

## The types, formation and research history of probiotic extracellular vesicles (pEVs)

Based on Gram staining of bacterial cells, pEVs can be categorized into those derived from Gram-negative and Gram-positive bacteria [39].

Early research on pEVs primarily focused on Gram-negative bacteria, which produce pEVs that can be classified into various species. Among these, the predominant type is the outer membrane vesicles (OMVs) [40]. OMVs are globular particles formed by the blistering of the outer membrane and cannot directly enter the cytoplasmic contents [41]. The envelope of Gram-negative bacteria consists of both an outer membrane and a cytoplasmic membrane, with the periplasmic space containing a layer of peptidoglycan (PG) between the two membranes [42]. The dissociation of the outer membrane from the underlying PG is believed to play a crucial role in the biogenesis of OMVs [43]. Additionally, physical stress caused by the accumulation of peptidoglycan fragments or misfolded proteins in the periplasm may lead to the bulging of the outer membrane, which is another proposed model for the generation of OMVs [44]. Changes in the cationic concentration of LPS can also result in local negative charge accumulation and repulsion between LPS molecules, leading to local deformation of the Gram-negative cell membrane, further contributing to OMV formation [45].

OMVs can be produced by various Gram-negative bacteria, including *Escherichia coli* Nissle 1917 (EcN) [46] and *Akkermansia muciniphila* [47]. Gram-negative bacteria also produce outer inner-membrane vesicles (OIMVs), which encompass both outer and inner membranes. Pérez-Cru et al. [48] first identified double bilayered membrane vesicles (MVs) in the study of *Shewanella vesiculosa* M7, coining the term OIMVs for these new extracellular vesicles. Certain antibiotics, including ciprofloxacin, can stimulate the formation of OIMVs through lysis, which may represent the primary pathway for the production [49]. It has also been suggested that autolysin can weaken the peptidoglycan layer of bacteria, causing the inner membrane to protrude into the peripheral space and form OIMVs, which contain cytoplasmic contents [50]. Furthermore, recent studies have identified novel Gram-negative extracellular vesicles. The degradation of the peptidoglycan layer due to phage invasion and the enzymatic action of endolysins leads to the formation of extracellular vesicles from bacterial lysis, termed explosive outer membrane vesicles (EOMVs) [50, 51]. Compared to OMVs, OIMVs and EOMVs contain a greater amount of cytoplasmic components [33].

At first, researchers believed that the thick peptidoglycan layer in the cell wall of Gram-positive bacteria could prevent the production of pEVs [52]. However, as research progressed, it was confirmed that Gram-positive bacteria are indeed capable of producing pEVs [53]. The pEVs produced by Gram-positive bacteria are termed cytoplasmic membrane vesicles (CMVs) due to the absence of an outer membrane. Enzymes that damage peptidoglycan are thought to play a significant role in the formation of CMVs. These peptidoglycan-damaging enzymes can induce a type of cell death known as “bubbling cell death”, which weakens the peptidoglycan layer and facilitates the production of CMVs [54]. Further research is necessary to understand the biogenesis of CMVs. Currently, various Gram-positive bacteria, including *Lactobacillus plantarum* [55], *Lacticaseibacillus rhamnosus* [56], *Lactobacillus johnsonii* [57], *Lactobacillus reuteri* DSM 17938 [58], and *Bacillus subtilis* [59, 60], have been confirmed to produce CMVs. Stress can lead to the lysis of Gram-positive bacteria in a process called “bubbling cell death”, which results in the generation of explosive cytoplasmic membrane vesicles (ECMVs) [33]. Notably, no specific stimulation of *Lactobacillus casei* is required, and the spontaneous induction rate of its prophages is sufficient to produce large amounts of ECMVs under normal culture conditions [61].

## Composition, extraction, and characterization of pEVs

pEVs comprise proteins, lipids, nucleic acids, and various small molecules [62]. They can be extracted using various methods, including centrifugation, ultrafiltration, commercial kits, lectin precipitation, and microfluidics [63, 64]. Their characterization can be broadly divided into two categories: physics and chemistry [65].

### Composition of pEVs

The composition of pEVs is influenced by the strain [31], the surrounding environment [66, 67], and the mechanisms of their formation [68].

Proteins are important components of pEVs. They contain a variety of proteins derived from parental bacteria, including outer membrane proteins, cytoplasmic proteins, and periplasmic proteins [69]. OMVs produced by Gram-negative bacteria originate from the outer membrane, which lacks cytoplasmic contents but contains a significant amount of periplasmic proteins [70, 71]. Proteomic analysis of OMVs produced by *Novosphingobium pentaromativorans* US6-1 indicated that outer membrane and periplasmic proteins are the main protein components of these OMVs [72]. This study also demonstrated that the outer membrane proteins in OMVs from *Novosphingobium pentaromativorans* US6-1 are derived from bacterial membrane-associated protein components. OIMVs, formed by protrusions from the inner membrane into the surrounding environment, and EOMVs, resulting from the explosive cleavage of bacteria, contain cytosolic contents [15]. Currently, the characterization of protein composition in CMVs produced by Gram-positive bacteria remains incomplete. It is generally accepted that most proteins in CMVs are cytoplasmic proteins. An analysis of CMVs produced from *Lactobacillus* indicated that 62%–82% of the identified proteins were intracellular and non-membrane-associated [73]. However, it has also been suggested that *Lactobacillus plantarum*-derived CMVs are rich in membrane-associated proteins [74]. CMVs produced by *Lactobacillus casei* BL23 primarily contain soluble proteins, including several metabolic enzymes, as well as proteins related to translation and transcription, with many chromosome-related proteins also present [61]. Studies of ECMVs and EOMVs have identified the presence of autolysins and endolysins [15, 19, 75]. pEVs contain proteins that promote bacterial colonization in the gut, compete with other bacteria, support cell survival, and regulate host immune functions [43]. In research on *Lactobacillus casei* BL23, a total of 103 proteins were identified using Liquid Chromatography Mass Spectrometry (LC-MS), with 13 specific to CMVs [76]. Among these, p40, p75, and LCABL_31160 are recognized as important proteins used by probiotics to protect the intestine, and they are enriched in CMVs [76]. Proteomic analysis of OMVs generated by *Escherichia coli* Nissle 1917 identified 192 highly reliable specific proteins, three of which are components of the iron uptake system [77]. These specific proteins facilitate the identification of EcN in the gut and enable competition with enteric pathogens that utilize similar siderophore-iron uptake systems [77]. Furthermore, proteomic analysis of OMVs from *Bacillus fragilis* revealed a substantial quantity of acidic hydrolytic enzymes, enhancing the ability of *Bacillus fragilis* to degrade polysaccharides and other glycoconjugates [78].

The lipid composition of the membrane determines the curvature and fluidity of the membrane, which can affect the biogenesis of pEVs. Consequently, the lipid composition of pEVs may differ from that of the cell membrane [19]. pEVs contain various lipids, including glycolipids and phospholipids, as well as lipopolysaccharides (LPS) [18]. OMVs produced by Gram-negative bacteria contain phospholipids and LPS, whereas CMVs produced by Gram-positive bacteria do not. The outer leaflet of OMVs from Gram-negative bacteria is primarily composed of LPS in addition to other lipids [79]. Although the lipid composition of pEVs generally reflects that of their parent bacteria, there are notable differences [15]. LC-MS was employed to compare the lipidomics of CMVs from *Lactobacillus plantarum* APsulloc 331261. The results identified 320 lipid species, of which 67 showed a significant increase and 19 exhibited a decrease, revealing significant differences from the parent bacteria [80]. Notably, lysophosphatidylserine (18:4) and phosphatidylcholine (32:2) were more than 21-fold enriched in CMVs [80]. Metabolomic analysis of CMVs produced by *Lactiplantibacillus plantarum* detected eicosatetraenoyl-glycerophosphate and various fatty acids that constitute the cytoplasmic membrane of bacteria, supporting the hypothesis that CMVs are derived from the cytoplasmic membrane of bacteria [81].

OMVs produced by Gram-negative bacteria were previously thought to contain DNA [82]. However, following the confirmation of OIMVs, it was proposed that most of the DNA resides within OIMVs rather than in OMVs [19]. At the same time, electron microscopy of OMVs (EOMVs) was shown to carry more DNA [19]. Therefore, it has been suggested that the presence of DNA in OMVs may result from contamination by OIMVs and EOMVs within the samples [19]. CMVs produced by Gram-positive bacteria also contain DNA [19]. RNA is generally viewed as a typical cytosolic component present in CMVs and EOMVs [19]. Biochemical analysis of CMVs derived from *Lactobacillus reuteri* indicated the presence of DNA, RNA, and protein [83]. When CMVs generated by *Lactobacillus reuteri* were treated with RNase, the results showed that RNase treatment had little effect on the amount of RNA content, the results showed that RNase treatment had little effect on the RNA content, indicating that the RNA was encapsulated within the CMVs [81].

**Fig. 1:**
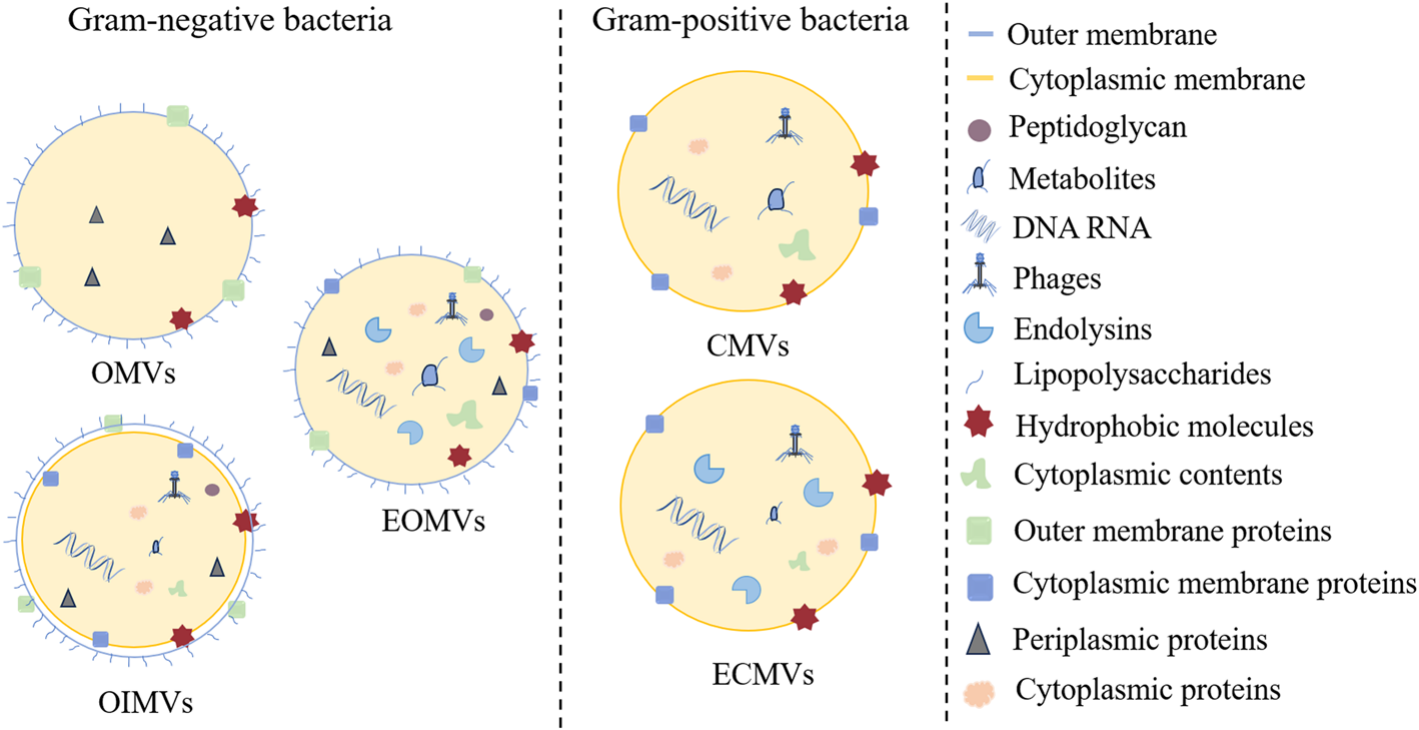
Composition of probiotic extracellular vesicles (pEVs) from Gram-negative and Gram-positive bacteria. pEVs contain proteins, nucleic acids, lipids, and various small molecules

pEVs contain a variety of small molecules, such as hydrophobic molecules, metabolites, and phages. Metabolomic analyses of pEVs have demonstrated that they contain specific metabolites that are selectively packaged, with composition depending on the bacterial source [84]. The metabolite content of individual strains of *Bacteroides thetaiotaomicron* is closely linked to their survivability, with the utilization of these metabolites enhancing bacterial survival in their ecological niches [85]. Additionally, these pEVs contain hydrophobic group-sensing molecules that modulate communication between bacteria [86]. The composition of pEVs is illustrated in Fig. 1.

### Extraction of pEVs

Ultracentrifugation is an effective method for extracting pEVs. It can be further categorized into differential ultracentrifugation and density gradient centrifugation. Differential ultracentrifugation allows for separation based on the density, size, and shape of particulate components in suspensions [87]. After precipitating larger and denser cellular debris and particles, the supernatant is further purified and centrifuged again to precipitate and wash the pEVs [87]. Density gradient centrifugation is another separation method that relies on size and density [88]. In this technique, a density gradient is created using a substance such as sucrose in a centrifuge tube [89]. The sample is then added to the top of the gradient, and centrifugal force is applied [90]. The particles in the sample increase in density at a distinct rate from the top to the bottom of the gradient, leading to their separation. Finally, the pEVs are collected through gradient-based collection [89].

Ultrafiltration is also one of the most common techniques used to extract pEVs [88]. Similar to conventional membrane filtration, the separation of suspended particles primarily depends on their size or molecular weight [91]. The filter retains particles larger than the molecular weight cutoff of the filter used, while smaller particles can pass through [91]. If pEVs are retained in the filter unit, they cannot be utilized for downstream analysis [92]. Residual contamination can be minimized by employing sequential filtration, using filters that range from larger to smaller pore sizes. Ultrafiltration is more convenient than ultracentrifugation and does not require specialized instrumentation [93]. However, shear forces during ultrafiltration may risk damaging the pEVs. This issue can be addressed by monitoring and regulating the transmembrane pressure [94].

Effective separation of pEVs can be achieved by altering their solubility or dissolution, prompting them to precipitate from biofluids. This process can be performed using water-free polymers like polyethylene glycol (PEG), which bind water molecules, allowing pEVs to precipitate through centrifugation [95]. This separation method is simpler and applicable to a wide range of starting volumes. However, it lacks selectivity, as it leads to the precipitation of both pEVs and other substances, such as extracellular proteins [94]. Typically, sample pretreatment, such as filtration, should be conducted before separation to ensure the purity of the precipitate when employing this method. Agglutinin can effectively replace PEG, as it specifically binds to carbohydrates on the particles, altering the solubility of pEVs and facilitating their precipitation and separation [96]. Additionally, ultracentrifugation pretreatment should be performed prior to precipitation to remove cell fragments and other carbohydrate-containing components [97].

Microfluidic-based separation of pEVs offers solutions to the challenges associated with traditional methods. By utilizing microfluidics, pEVs can be separated based on both physical and biochemical properties, offering advantages such as high efficiency and low initial volume requirements [98]. Continuous advancements in separation mechanisms now allow for the separation of pEVs based on their acoustic, electrophoretic, and electromagnetic properties. One notable microfluidic separation technique is the use of acoustic nanofilters [99]. In this method, a matrix containing pEVs and other extracellular components is injected into a chamber exposed to ultrasound, which exerts radiative forces on the particles. Particles of varying sizes and densities respond differently to these forces, with larger particles experiencing stronger radiative forces and migrating more rapidly toward the pressure nodes [99]. Ultrasound-based separation can accommodate particles across a wide range of sizes [99]. While this method is still in development, it shows great promise for future applications.

Despite the existence of various methods for separating pEVs, differential ultracentrifugation remains the gold standard. This technique effectively separates larger and denser particles based on density, size, and shape, making it particularly suitable for efficient separation [94].

### Characterization of pEVs

In the early stages of research, the characterization of pEVs primarily focused on protein concentration [62, 94]. However, different subtypes of pEVs may exhibit varying protein profiles, and the protein concentration in isolated pEVs is often overestimated [94]. As research methods for pEV characterization have advanced, the analysis techniques have also been updated and refined. These methods can now be categorized into physical and chemical analysis techniques [100]. Physical analysis of pEV size and concentration predominantly include nanoparticle tracking analysis (NTA) [101], dynamic light scattering (DLS) [102], electron microscopy [103], and tunable resistance pulse sensing (tRPS) [104]. Chemical analyses of the composition of pEV contents mainly involve staining methods [105], immunoblotting [106], and proteomics [107].

NTA utilizes the principles of light scattering and Brownian motion to assess the size and concentration of pEVs [108]. The behavior of particles like pEVs is influenced by the surrounding molecules, leading them to exhibit irregular Brownian motion. In this technique, laser light is directed onto the sample, and the trajectory of the Brownian motion is observed using an optical microscope. The NTA software estimates the sizes and concentrations of the particles based on their motion [109]. Dynamic light scattering (DLS) operates similarly to NTA but relies on fluctuations in the intensity of scattered light to determine particle size [110]. DLS typically requires a very small sample, necessitating less extensive parameter optimization [102]. However, because detecting scattered light from small particles can be challenging, DLS often skews data towards larger particle sizes when analyzing non-homogeneous mixtures [102, 111]. Electron microscopy provides a means to directly observe the morphology of pEVs. This method employs an electron beam to generate high-resolution images of submicron particles and is commonly performed using both transmission electron microscopy (TEM) and scanning electron microscopy (SEM) [112]. The primary distinction between these two methods lies in their electron detection techniques [112]. Tunable resistance pulse sensing (tRPS) employs a non-conducting nanomembrane to partition a fluid cell into two sections: a suspension and a particle-free electrolyte [113]. When an electrical potential is applied to move the particles across the nanomembrane, the current between the sections is interrupted, resulting in a resistive pulse [114]. The duration of this resistive pulse is correlated the size of the particles [114].

Immunodetection methods are used for visualizing labeled proteins and identifying target proteins to assess the purity of isolated pEVs. Flow cytometry can be employed for the immunophenotyping of pEVs [115]. Additionally, various proteins within pEVs can be isolated using Western blotting; after transfer, these proteins are incubated with antibodies specific to the target protein before detection [94]. However, this approach does not facilitate the observation of intact vesicles [116].

**Fig. 2:**
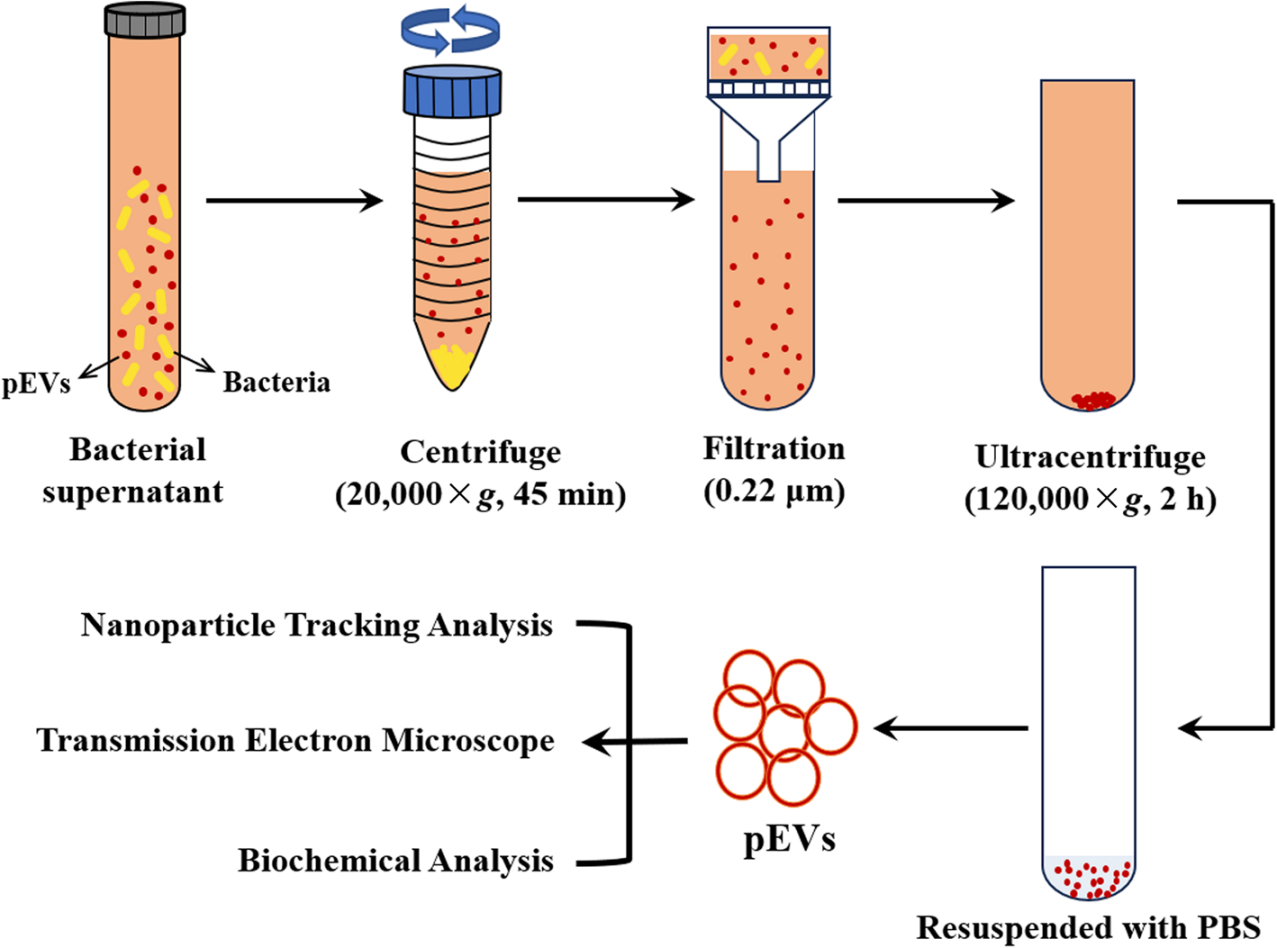
Isolation and characterization of pEVs. The pEVs in the solution are separated using differential centrifugation, rinsed with PBS, and then analyzed using NTA, transmission electron microscopy, and biochemical assays

Currently, differential centrifugation is commonly employed technique for extracting pEVs, while methods such as NTA, TEM, and biochemical analyses are utilized to study their size, concentration, and composition [103, 117], as illustrated in Fig. 2.

## Significance of in-depth study of pEVs in livestock and poultry production

Currently, many diseases affecting livestock and poultry are linked to microorganisms [118]. Among them, piglet diarrhea is primarily caused by disruptions in intestinal flora and compromised intestinal barriers [119]. Early weaned piglets are particularly susceptible to enterotoxigenic *E. coli* (ETEC) infections, which result in diarrhea and account for the deaths of up to 50% of piglets worldwide each year [119]. During breeding, antibiotics are often employed to treat diseases caused by microorganisms; however, the extensive use of antibiotics has led to the emergence of antibiotic resistance in livestock and poultry. This scenario poses significant challenges to the sustainable development of the breeding industry and human health [120–122]. Probiotics present an effective alternative to antibiotics by reshaping intestinal flora, alleviating and treating livestock and poultry diseases, and circumventing antibiotic resistance and contamination [123]. For instance, *Bacillus licheniformis* has been shown to reduce the incidence of diarrhea and decrease the relative abundance of intestinal *E. coli* [124]. Similarly, *Bifidobacterium lactis* and *Lactobacillus rhamnosus* inhibit the adhesion of pathogens such as *Salmonella*, *Clostridium*, and *E. coli* to porcine intestinal mucus [125]. Probiotics are generally involved in their interactions with the host by secreting bioactive substances, thus enhancing the integrity of the intestinal epithelial barrier [11]. Studies conducted over the past decade indicate that the health-promoting effects of probiotics are largely dependent on the production of pEVs [10]. Therefore, an in-depth exploration of the significance and mechanisms of pEVs in livestock and poultry breeding can further enhance the beneficial roles of probiotics, ultimately promoting the sustainable and healthy development of animal husbandry.

## Effects of pEVs on the intestinal barrier and potential mechanisms

The intestinal barrier consists of biological, immune, chemical, and physical components, all of which contribute to its integrity-crucial for livestock physiology and homeostasis [126, 127]. An intact intestinal epithelial barrier effectively prevents luminal microorganisms and antigens from entering the bloodstream [128, 129]. Disruption of the self-renewal function of intestinal epithelial cells (IECs) can lead to intestinal barrier dysfunction, resulting in increased intestinal permeability [130]. This heightened permeability allows direct contact between host cells and intestinal luminal contents, including commensal bacteria and foreign pathogens [129, 130]. Consequently, this interaction triggers intestinal mucosal immune responses, potentially leading to inflammation, allergies, and metabolic diseases in livestock and poultry [129]. Conversely, the intestinal epithelial barrier can hinder effective communication between probiotics and the host [131]. To address this, probiotics regulate host functions by secreting bioactive substances, such as pEVs [131]. Researchers have conducted in-depth studies on this phenomenon, demonstrating that pEVs can enter host cells, including immune and IECs, through endocytosis to exert their effects [11, 132]. The specific impacts of pEVs on each type of barrier and the potential mechanisms involved are detailed below.

### Intestinal biological barrier

Reduced intestinal barrier function is primarily manifested by alterations in the composition or abundance of microorganisms colonizing the gut, resulting in dysbiosis characterized by an unbalanced intestinal microbiota, decreased microbial diversity, and an increased presence of pathogenic bacteria [133]. These changes lead to a decline in intestinal function and may trigger various intestinal diseases [133]. In piglets, intestinal dysbiosis is predominantly reflected in the altered structure of the bacterial community, marked by a deficiency of *Lactobacillus* and an excess of *E. coli* [119, 134, 135]. pEVs can support the growth and colonization of beneficial microorganisms, thereby aiding livestock and poultry in establishing a balanced intestinal microbiota [20]. pEVs contain adhesion factors that promote the colonization of intestinal probiotics and harbor polysaccharide-degrading enzymes [136]. These enzymes can break complex polysaccharides to produce short-chain carbohydrates or can break complex polysaccharides to produce monosaccharides, providing nutrients for the intestinal probiotics [136]. Analysis of biomolecular interactions has demonstrated that pEVs contain numerous cytoplasmic proteins that bind to porcine gastric mucins, such as elongation factor Tu, phosphoglycerate kinase, and heat shock proteins. Consequently, pEVs enhance the colonization of probiotics by transporting bacterial adhesion factors to the extracellular environment [137]. Furthermore, *Akkermansia muciniphila*-derived pEVs selectively promote the colonization of beneficial bacteria through membrane fusion, thereby maintaining a balanced intestinal microbiota [20].

pEVs derived from *Lactobacillus rhamnosus* GG have been shown to restore deficits in alpha and beta diversity induced by dextran sulfate sodium salt [138]. Similarly, pEVs produced by *L. plantarum* Q7 alleviated inflammation by modulating the intestinal microbiota in a mouse model of DSS-induced colitis, increasing the abundance of *Akkermansia*, *Bifidobacteria*, *Muribaculaceae*, and *Lactobacillus*, while decreasing that of *Proteobacteria*, *Deferribacteria*, and *Epsilonbacteraeota* [139]. Additionally, the pEVs of *Bifidobacterium* contain mucin-binding proteins that facilitate their colonization of the intestinal mucosa [137]. For instance, *B. ovatus*-derived pEVs carry inulin-degrading enzymes that hydrolyze inulin, generating nutrients that facilitate the growth of *Bacteroides* species unable to metabolize inulin [25]. Moreover, pEVs can transfer antimicrobials to competing pathogenic bacteria, causing structural damage, dysfunction, and cell death, ultimately leading to the elimination of these competing bacteria [44]. pEVs derived from *L. rhamnosus* GG exhibit bactericidal effects against various pathogens, including *Pseudomonas*, *Salmonella*, *Clostridium*, and *E. coli* B-44 [140]. Proteomic analysis of pEVs has revealed a significant enrichment in bacteriocins [73]. These bacteriocins can be utilized by pEVs to eliminate competing bacteria while also protecting themselves from proteases and inactivating molecules present in the gut [15, 73]. Bacteriocins have the capability to inhibit or kill several harmful bacteria, including *Staphylococcus aureus*, *L. monocytogenes*, and *P. aeruginosa* [141].

Antibiotics and host defense peptides (HDPs) present considerable threats to the survival of gut bacteria within the host [142]. pEVs can interact with antibiotics and HDPs to reduce their concentrations, thereby safeguarding sensitive bacterial strains. For example, pEVs can bind to membrane-targeted antibiotics such as polymyxins and daptomycin [44]. *Bacteroides*-derived pEVs possess β-lactamases that hydrolyze β-lactam antibiotics, enhancing the survival of intestinal commensal bacteria [143]. Additionally, pEVs derived from *L. plantarum* have been shown to maintain intestinal homeostasis in vitro by increasing the expression of antimicrobial peptides, such as histone B and RegIIIγ, in the colonic epithelial cell line Caco-2 [74].

### Intestinal immune barrier

pEVs are key components in immune signaling [144]. Studies have demonstrated that pEVs influence various immune signaling pathways, including the intracellular nucleotide oligomerization domain 1 (NOD1), NF-κB, TLR2, ERK, and NLRP3 pathways, as well as the secretion of immune cytokines. These pathways enhance the immune function of the intestinal tract in animals, helping to prevent intestinal inflammation [57, 83, 144–150]. Peptidoglycan in pEVs can activate NOD1 receptors in IECs, thereby enhancing innate intestinal immunity [145]. pEVs derived from *E. coli* Nissle 1917 (EcN) specifically act on the NOD1 signaling pathway to improve intestinal function, positively influencing intestinal homeostasis [145, 146]. DSS can upregulate the expression of *TLR4* and *MyD88* genes, as well as the p65 and p-p65 proteins in the NF-κB signaling pathway, leading to increased expression of the Nod-like receptor family in the NLRP3 signaling pathway and promoting pro-inflammatory factor expression [138]. Furthermore, pEVs from *L. rhamnosus* and *L. kefiri* exhibit anti-inflammatory effects by suppressing the overexpression of TNF-α, IL-1β, IL-6, and IL-2 in DSS-treated intestinal tracts, primarily through the inhibition of the NF-κB signaling pathway [138, 147]. The pEVs from *Propionibacterium freudenreichii* CIRM-BIA 129 rely on surface layer proteins (SlpB) to interact with NF-κB and inhibit IL-8 secretion [148]. Additionally, pEVs from *L. murinus* reduce the overexpression of pro-inflammatory factors in the intestine by enhancing the expression of TLR2 [149]. pEVs from *L. john* improve the host’s intestinal barrier function by inhibiting extracellular signal-regulated kinase (ERK) expression, promoting M2 macrophage polarization, and obstructing pathways such as NLRP3 [57]. In vitro studies indicate that pEVs derived from *L. reuteri* and *L. paracasei* inhibit the expression of pro-inflammatory factors while promoting anti-inflammatory factors like IL-10 and TGF-β, thereby alleviating intestinal inflammation [83, 150]. Furthermore, pEVs of *L. paracasei* have been shown to inhibit the activation of inflammatory cell lines such as Caco-2, iNOS, NF-κB, and NO within signaling pathways, effectively suppressing the LPS-induced inflammatory response in human colonic adenocarcinoma HT-29 cells [150]. The anti-inflammatory effects of *L. paracasei*-derived pEVs were diminished when endoplasmic reticulum stress inhibitors were introduced, indicating their relevance to endoplasmic reticulum stress [150]. In conclusion, pEVs modulate immune signaling pathways, regulate the secretion of anti-inflammatory and pro-inflammatory factors, and significantly influence intestinal immune function.

It has been demonstrated that pEVs can activate immune cells such as macrophages, intestinal regulatory T cells (Tregs), dendritic cells, and neutrophils [15, 83, 132, 151–157]. This activation occurs through two main mechanisms. First, pEVs carry microbe-associated molecular pattern (MAMP) molecules that can be recognized by specific pattern recognition receptors expressed by host epithelial and immune cells [44, 158]. Upon internalization by IECs, pEVs activate the cytoplasmic receptor NOD1 [15]. This activation triggers the secretion of immune effector factors, leading to the production of multiple cytokines and co-stimulatory molecules. This process is classified as indirect activation [15]. Second, pEVs can penetrate the epithelial barrier to reach the small intestinal mucosa, where they activate intestinal immune cells [15]. Intestinal macrophages serve as central immunoregulatory cells in the intestine [159]. They sense various signals in the intestinal environment, leading to changes in their phenotype and function, which enable them to perform corresponding immune functions [160]. Macrophages are also crucial participants in the intestinal inflammatory response, producing both pro-inflammatory and anti-inflammatory cytokines [161]. Furthermore, pEVs can be taken up by macrophages, exerting an anti-inflammatory effect [151]. By modulating immune plasticity through macrophages, pEVs play a role in maintaining the integrity of the intestinal barrier [151]. For example, pEVs produced by *Pediococcus pentosaceus* effectively alleviated DSS-induced acute colitis by activating TLR2 and promoting the polar differentiation of macrophages into M2-like macrophages [152]. Additionally, researchers treated macrophages with pEVs from *L. reuteri* and then co-cultured them with splenic lymphocytes. This resulted in a downregulation of IFN-γ and IL-17 expression in the splenic lymphocytes, effectively inhibiting their pro-inflammatory response, while the expression of CD25, CTLA-4, and LAG-3 was upregulated [83]. Therefore, CD4^+^CD25^+^ cells may also be involved in the immunomodulatory effects mediated by *L. reuteri*-derived pEVs [83]. Certain pEVs exhibit Treg-inducing effects as well. For instance, pEVs released by *Bacteroides fragilis* deliver polysaccharides that are taken up by dendritic cells (DCs) upon entry into the body. This interaction with TLR2 helps prevent intestinal inflammation [132, 153]. These polysaccharides also promote the production of Tregs and anti-inflammatory cytokines, which assist in regulating the immune response [132]. pEVs derived from *L. rhamnosus* and *Bifidobacterium* enhance the development of Tregs and tolerance-promoting dendritic cells by interacting with the C-type lectin receptor and TLR on DCs [154, 155]. Similarly, pEVs from *B. vulgatus* promote DC hemiactivation and enhance immune system silencing, thereby attenuating colitis in the host [156]. Moreover, pEVs from *Escherichia coli* Nissle (EcN) and *B. thetaiotaomicron* can help resist pathogenic infections in the gut by activating DCs to initiate a Th1-type response [153, 157]. Finally, pEVs regulate neutrophils in a dose-dependent manner, impacting memory-like inflammatory responses [162]. Studies have shown that fecal-derived pEVs directly mediate adaptive immune responses [163]. A low dose of pEVs (1 ng/mL) significantly enhanced pro-inflammatory sensitivity in neutrophils, while higher doses resulted in diminished responses to LPS stimulation [163]. In summary, pEVs modulate intestinal barrier function by affecting intestinal macrophages, Tregs, dendritic cells, and neutrophils.

### Intestinal chemical barrier

The intestinal chemical barrier consists of mucus, digestive juices, antimicrobial peptides (AMPs), and other antimicrobial substances secreted by the intestinal mucosal epithelium [164, 165]. This barrier separates intestinal bacteria from the intestinal epithelium and influences their survival capacity [33]. Several studies have shown that pEVs positively affect the function of the chemical barrier, enhancing the ability of probiotics to survive [20, 74]. The mucus on the surface of the intestinal epithelium serves as the gut’s primary defense against intestinal microbiota and pathogenic bacteria [165]. Goblet cells, predominantly found in the colon of livestock and poultry, are the main epithelial cells responsible for secreting mucus in the intestines [166]. Accumulating evidence suggests that processes regulating intestinal epithelial fluid absorption and secretion, closely related to intestinal health, can be influenced by several pEVs [20]. For instance, the internalization of *A. muciniphila*-derived pEVs leads to a significant increase in the number of Goblet cells and stimulates mucus production, resulting in a thicker mucus layer in the intestinal lumen that offers resistance to pathogenic bacteria [20]. Antimicrobial peptides secreted by epithelial and Paneth cells can help prevent bacteria from penetrating the internal mucus layer [167]. Furthermore, pEVs can induce the expression of intestinal AMPs, enhancing the chemical barrier. Studies have shown that *Lactobacillus*-derived pEVs promote the expression of the AMP REG3G. As a C-type lectin, REG3G enhances the capacity of the intestinal chemical barrier [74]. Clearly, pEVs stimulate enterocytes, leading to increased AMP and mucus production, and contribute to the enhanced impenetrability of the intestinal chemical barrier [74].

### Intestinal physical barrier

Intestinal epithelial cells and the connectivity complex, which includes tight junctions (TJs), adherens junctions, gap junctions, and desmosomes, form the intestinal physical barrier [168–170]. IECs are closely linked to adjacent cells through cell-cell junctions, which effectively prevent bacterial invasion [171]. They can sense microbial signals through PRRs [171]. Upon activation, IECs enhance the intestinal barrier, thereby protecting host tissues [171]. Tight junctions are highly complex macromolecular protein structures located in the apical region of the junctional complexes, overlaying the polarized epithelium. TJs are formed by transmembrane proteins such as occludin, claudin, and tricellulin, as well as adhesion molecules that establish intercellular connections [172]. pEVs can influence intestinal health by acting directly on the IECs or by regulating the formation of TJs between neighboring IECs [131, 146, 173–179]. *Bacteroides*-derived pEVs contain human keratinocyte growth factor 2 (KGF2), which aids in the repair of IECs in animals with colitis [173]. pEVs derived from *L. reuteri* DSM 17938 and BG-R46L can attenuate leakage induced by enterococcal-producing *E. coli* after being taken up by IECs [174]. Additionally, pEVs released by *A. muciniphila* can directly reduce intestinal permeability by penetrating IECs and promoting the expression of TJ proteins [175]. These pEVs act on AMP-activated protein kinase (AMPK) to upregulate the animal’s TJ closure proteins, including ZO-1, occludin-1, and claudins-1 [175]. Furthermore, EcN-derived pEVs have been shown to upregulate ZO-1 and claudin-14 while downregulating claudin-2 in IECs via tcpC [176, 177]. This process enhances the TJs between IECs [178], promotes goblet cell activity by stimulating IL-22 expression, and activates Trefoil Factor 3 (TFF-3) in cup cells [131]. Additionally, this mechanism increases the expression of antimicrobial factors such as β-defensin-2 and positively regulates the intestinal epithelial barrier by downregulating the expression of matrix metalloproteinase-9 (MMP-9), which disrupts the TJ [146, 179]. Evidently, pEVs play a crucial role in regulating IECs and TJs, thereby contributing to the reinforcement of the intestinal physical barrier.

### Interaction between pEVs and various parts of the intestinal barrier

**Fig. 3:**
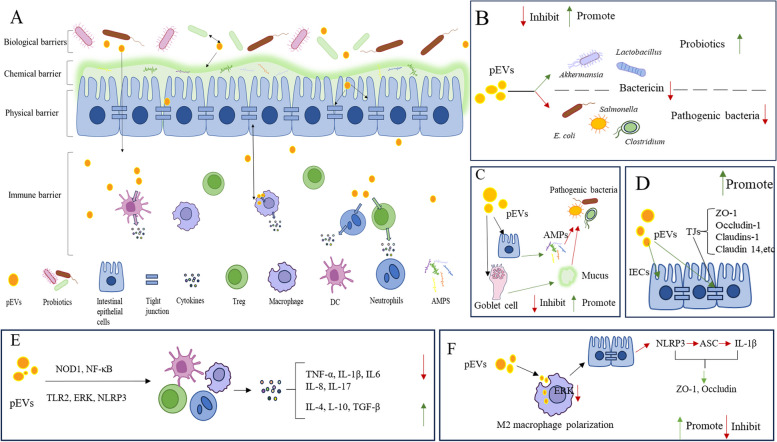
An illustration of the mechanisms through which pEVs influence various intestinal barriers. **A** A general overview of the effects of pEVs on the intestinal barrier. **B** Regulation of the biological barrier of the intestine by pEVs. **C** Effects of pEVs on the chemical barriers. **D** Modulation of the physical barriers by pEVs. **E** pEVs’ influence on the immune barrier. **F** pEVs promote interactions between the intestinal immune and physical barriers

There is an inter-regulatory effect among the various components of the intestinal barrier, as well as a regulatory influence of pEVs on these barriers [180]. Macrophages and IECs are interdependent and can interact with one another [180]. Recent studies have shown that pEVs secreted by *Lactobacillus mucosae* counteract colitis-induced damage to the intestinal physical barrier by modulating macrophage phenotypes [151]. Specifically, they promote M2-type macrophage polarization, inhibit M1-type macrophage polarization, and suppress the expression of inflammatory regulatory proteins such as NF-κB and AKT [151]. Additionally, pEVs can regulate the innate immunity of IECs by interacting with Toll-like receptor 2 (TLR2), which plays a crucial role in recognizing and signaling innate immune cells in response to molecular patterns associated with bacterial pathogens [149]. *L. murinus*-derived pEVs mitigate deoxynivalenol-induced intestinal barrier disruption by activating TLR2, thereby promoting the conversion of anti-inflammatory macrophages to the α-smooth muscle actin phenotype [149]. This process decreases levels of pro-inflammatory cytokines (such as IL-1β and TNF-α), increases the secretion of anti-inflammatory cytokines, and enhances the expression of TJ proteins [149]. Evidently, pEVs play a vital role in maintaining intestinal homeostasis by enhancing the interaction between the immune barrier (macrophages) and the physical barrier (IECs). Overall, pEVs contribute to the regulation of all intestinal barriers through a unique approach. An interactive regulatory effect exists among these barriers, as illustrated in Fig. 3. Consequently, the role of pEVs in the intestinal health of livestock and poultry is significant and cannot be overlooked.

## Conclusions and perspectives

The study of pEVs has advanced significantly. This overview highlights the composition, extraction, and characterization of pEVs, with a focus on the potential mechanisms by which pEVs influence various intestinal barriers. Understanding these mechanisms is essential for gaining insights into how pEVs contribute to maintaining the intestinal health of livestock and poultry, thereby enhancing their growth efficiency and immune responses. This knowledge has profound implications for the sustainable development of the livestock industry and improved animal welfare. While some mechanisms of pEVs’ action on intestinal barriers have been identified, others remain to be explored. Most current research has examined pEVs in their entirety when investigating their role in regulating intestinal barrier function, leaving the specific components and the associated mechanisms still unclear. There is an urgent need to identify these components to provide a theoretical basis for the effective utilization of pEVs. Future practical applications of pEVs to enhance the intestinal health of livestock and poultry must address the challenges of increasing pEV production and maintaining their long-term efficacy. In conclusion, there is still much to learn about pEVs, their mechanisms of action, and their potential applications in animal production.

## Abbreviations

AMP: Antimicrobial peptide
DLS: Dynamic light scattering
IECs: Intestinal epithelial cells
KGF2: Keratinocyte growth factor 2
MAMPs: Microbe-associated molecular patterns
NOD 1: Nucleotide oligomerization domain 1
NTA: Nanoparticle tracking analysis
pEVs: Probiotic extracellular vesicles
PEG: Polyethylene glycol
PRR: Pattern recognition receptor
TJ: Tight junction
tRPS: Tunable resistance pulse sensing
